# Association between physical activity and risk of premenstrual syndrome among female college students: a systematic review and meta-analysis

**DOI:** 10.1186/s12905-024-03147-3

**Published:** 2024-05-23

**Authors:** Hongchang Yang, Yuhan Ma, Ying Wang, Chengjie Fu, Wanduo Liu, Wenchao Li

**Affiliations:** 1 Department of Physical Education, Hohai University, Nanjing, Jiangsu People’s Republic of China; 2 College of Geography and Remote Sensing, Hohai University, Nanjing, Jiangsu People’s Republic of China; 3 College of Physical Education and Training, Capital University of Physical Education And Sports, Beijing, People’s Republic of China

**Keywords:** Physical activity, Premenstrual syndrome, College students, Meta-analysis

## Abstract

**Background:**

This study aimed to analyze the relationship between physical activity and the risk of premenstrual syndrome among college students.

**Methods:**

Eligible studies were searched from the PubMed, Web of Science, and Embase databases. The link between physical activity and the risk of premenstrual syndrome was evaluated using odds ratio (OR) and 95% confidence interval (CI). The heterogeneity of the included studies was tested and their sources were explored by subgroup analysis. A sensitivity analysis was performed to assess the effect of a single study on the pooled results. The included studies were evaluated for publication bias. Five moderate-quality studies were included in this meta-analysis.

**Results:**

Physical activity levels were negatively associated with risk of premenstrual syndrome among college students (OR [95%CI] = 1.46 [1.09, 1.96], *P* = .011). The pooled results were not influenced after being stratified by the study region and whether multi-factor correction was performed or not. Publication bias was not observed in the included studies.

**Conclusion:**

A high level of physical activity is dramatically associated with a reduced risk of premenstrual syndrome among female college students.

**Supplementary Information:**

The online version contains supplementary material available at 10.1186/s12905-024-03147-3.

## Introduction

Premenstrual syndrome is a prevalent condition faced by women of reproductive age that negatively affects their emotions and performance [1, 2]. It refers to multiple distressing symptoms that occur periodically during the late luteal phase of the menstrual cycle (7–14 days before menstruation), including abdominal pain, low back pain, mood swings, irritability, changes in appetite, anxiety, anger, fatigue, and headache [3, 4]. These somatic and psychological symptoms peak within a week before menstruation and subside with the onset of menstruation [5, 6]. It is reported that 20–40% of menstruating women are affected by premenstrual syndrome [7].

College students are in a transitional phase from adolescence to adulthood, and mental illness is likely to occur during this period [8]. Female college students are particularly susceptible to premenstrual syndrome because it not only impacts their daily lives and academic performance but also their psychomotor functions due to changes in cognitive-emotional processes [9, 10]. A previous meta-analysis showed that the prevalence of premenstrual syndrome was 50.3% among college students [11]. A cross-sectional study showed that the prevalence of premenstrual syndrome was 62.7% in 300 college students in Puducherry [12]. Premenstrual syndrome can result in higher rates of absenteeism, impaired academic performance, lack of engagement in physical activities, poor quality of life, and substantial social distress among students [13, 14]. Given the high prevalence of premenstrual syndrome in college students, it is crucial to identify the key factors responsible for mitigating the symptoms, which can help prevent severe symptoms and potentially unnecessary medical consultations.

As the etiology of premenstrual syndrome is largely unknown, different treatments have been proposed [15]. Due to the side effects of drug treatments (such as antidepressant tablets), alternative non-pharmacological treatments, particularly physical activity, have emerged as significant factors for promoting physical and mental health among women [16]. Multiple studies investigating the association between physical activity and premenstrual syndrome among female college students have reported inconsistent findings. For instance, moderate or high physical activity is associated with milder symptoms of premenstrual syndrome [12, 17, 18]. However, Sahin et al. suggested that physical activity was not sufficient to yield beneficial effects on premenstrual syndrome [19]. Considering this research controversy, the impact of physical activity on premenstrual syndrome among female college students should be further explored.

Herein, we performed a meta-analysis to investigate the association between physical activity and premenstrual syndrome among female college students based on multiple available studies. Our findings provide evidence to inform future interventions to mitigate premenstrual syndrome among female college students.

## Methods

### Search strategy

Using a pre-established search strategy, relevant studies were retrieved from three electronic databases: PubMed, Web of Science, and Embase. The keywords for the search included “premenstrual syndrome,” “premenstrual dysphoric disorder,” “university,” “students,” “exercise,” and “physical activity.” The search keywords in different categories were combined with “AND” and those in the same category were associated with “OR”. Subject and free words were combined for the search, and the search steps were determined according to the characteristics of each database (Supplementary Tables 1–3). The search time was up to October 7, 2023 with no language limitation set. Additionally, references from the included studies and relevant reviews were screened to obtain additional studies suitable for meta-analysis.

### Study selection

The inclusion criteria for study selection were as follows: (1) the subjects were female college students; (2) the study had an observational design (i.e., case-control, cross-sectional, and cohort studies); (3) the study reported the relationships between physical activity levels and the risk of premenstrual syndrome, with no specific restrictions on the methods used to measure physical activity or the criteria used for grouping; and (4) the association strength was evaluated as odds ratio (OR) and 95% confidence interval (CI) or could be calculated using other available data.

The exclusion criteria were as follows: (1) non-authoritative sources such as comments, reviews, and conference abstracts; (2) intervention studies; and (3) duplicate publications or multiple studies that had the same data, except for the study providing the most comprehensive research data.

### Data extraction and quality assessment

According to the inclusion and exclusion criteria, the study selection was independently completed by two investigators. Then, two investigators independently extracted the data according to a predesigned table. Extracted data included the following: study type, publication year, first author, basic characteristics of the study participants (sex, age, and sample size), measurement criteria for premenstrual syndrome, and definition and grouping criteria of physical activity. After the completion of data extraction, the extraction table was exchanged and disagreements were resolved via discussion.

The Newcastle–Ottawa Scale [20] was used to evaluate the quality of case-control and cohort studies. The scale consisted of three aspects: comparability, selection of study participants, and exposure. It consisted of eight scoring items, with a maximum score of 9. A score ranging from 7 to 9 indicated high quality; from 4 to 6, moderate quality; and less than 4, low quality.

The Agency for Healthcare Research and Quality (AHRQ) scale was used to assess the quality of cross-sectional studies. This scale comprised 11 evaluation items, each of which was scored 1 for “yes” and 0 for “no” or “unclear” responses. Scores of 8–11, 4–7, and 0–3 represented high, moderate, and low quality, respectively.

### Statistical analysis

Physical activity was divided into high and low levels, and the relationships between physical activity levels and the risk of premenstrual syndrome were evaluated using OR with 95%CI as the effect size measure. Because of the remarkable differences in the design of the included studies, the data were pooled using the random effects model in the meta-analysis. Heterogeneity was tested using Cochran’s Q test and I^2^ test [21]. If *P* < .05 and/or I^2^ > 50%, there was significant heterogeneity among the studies. Subgroup analysis was performed according to the study region and whether multi-factor correction was performed. The impact of a single study on the pooled results was explored by a sensitivity analysis using the leave-one-out method [22]. Publication bias in the included studies was evaluated using Egger’s test and a funnel plot [23]. All statistical analyses were performed using Stata 12.0 software (StataCorp, College Station, TX, USA).

### Ethical review

Ethical approval and patient consent were not required because the meta-analysis and bioinformatic analyses were based on published research and public database.

## Results

### Literature search results

**Fig. 1:**
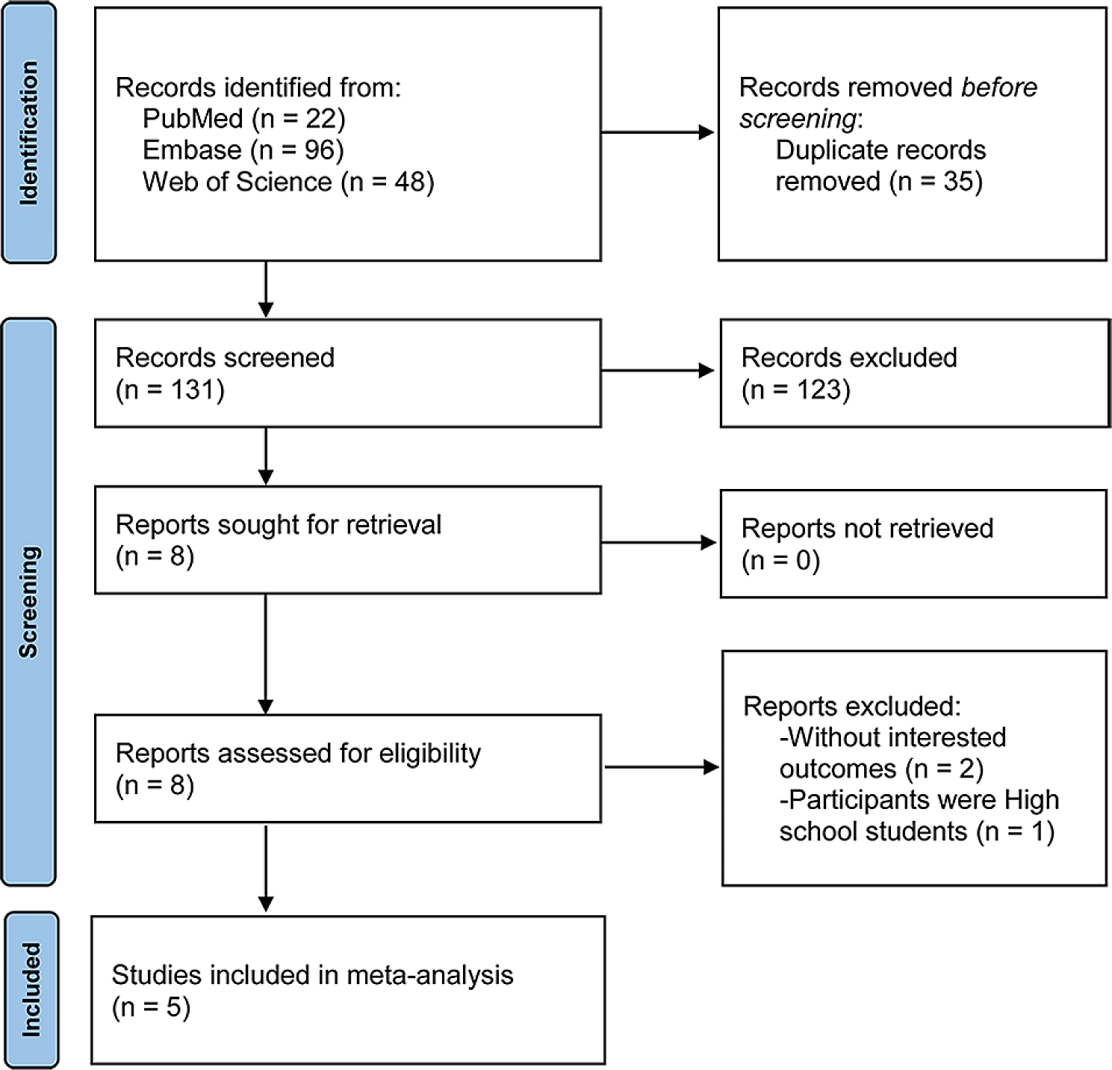
Literature search process and results

The literature search yielded 166 results (PubMed: 22, Embase: 96, and Web of Science: 48), of which 35 duplicates were removed. Based on title and abstract screening, 123 records were excluded. By further full-text screening, 5 eligible publications [12, 17, 18, 24, 25] were included in the meta-analysis. The literature search process and results are shown in Fig. 1.

### Study characteristics and quality evaluation results

The five included studies were published between 2010 and 2023, and all were cross-sectional studies conducted in Asia. The sample sizes in these studies ranged from 164 to 381 participants, with a total of 1316 participants, all of whom were college students. Except for the study by Kawabe et al [18], which used convenience sampling, the studies did not report the sampling methods of the study subjects. Study characteristics are presented in Table 1.

**Table 1:** Characteristics of the 5 included studies

Study	Country	Duration	Assessment of PMS	Sample Size	PMS, Yes/No	Age, years	Type of PA	Comparison	OR (95%CI)	Multivariate adjusted
Balaha, MH 2010	Saudi Arabia	2009.06-12	ACOG PMS diagnostic criteria	250	89/161	19.86 ± 1.13	Regular PA	No vs. Yes	0.914 (0.823, 1.016)	Yes
Bhuvaneswari, K 2019	India	NR	Shortened Premenstrual Assessment Form	300	188/112	18–22	Regular PA	No vs. Yes	6.600 (3.931, 11.082)	No
Kawabe, R 2022	Japan	2016.08-09	ACOG PMS diagnostic criteria	381	344/37	20.4 ± 1.2	Total PA	< 3000 vs. ≥3000 METs	1.031 (1.010, 1.075)	Yes
Shah, RS 2020	India	NR	Premenstrual Symptoms Screening Tool	164	31/133	18–24	Regular PA	No vs. Yes	1.061 (0.448, 2.517)	No
Shi, Y 2023	China	2021.10-2022.03	Premenstrual Symptoms Screening Tool	221	148/73	21.69 ± 2.50	MVPA	Low vs. High	1.639 (1.220, 2.203)	Yes

ACOG, American College of Obstetrics and Gynecology; MVPA, moderate-to-vigorous Physical activity; NR, not reported; PA, Physical Activity; PMS, premenstrual syndrome

As all the included studies were cross-sectional, quality evaluation was conducted using the AHRQ. The results showed that the AHRQ scores of the included studies ranged from 5 to 7 (Supplementary Table 4), indicating moderate methodological quality.

### Physical activity was negatively linked to the risk of premenstrual syndrome

**Fig. 2:**
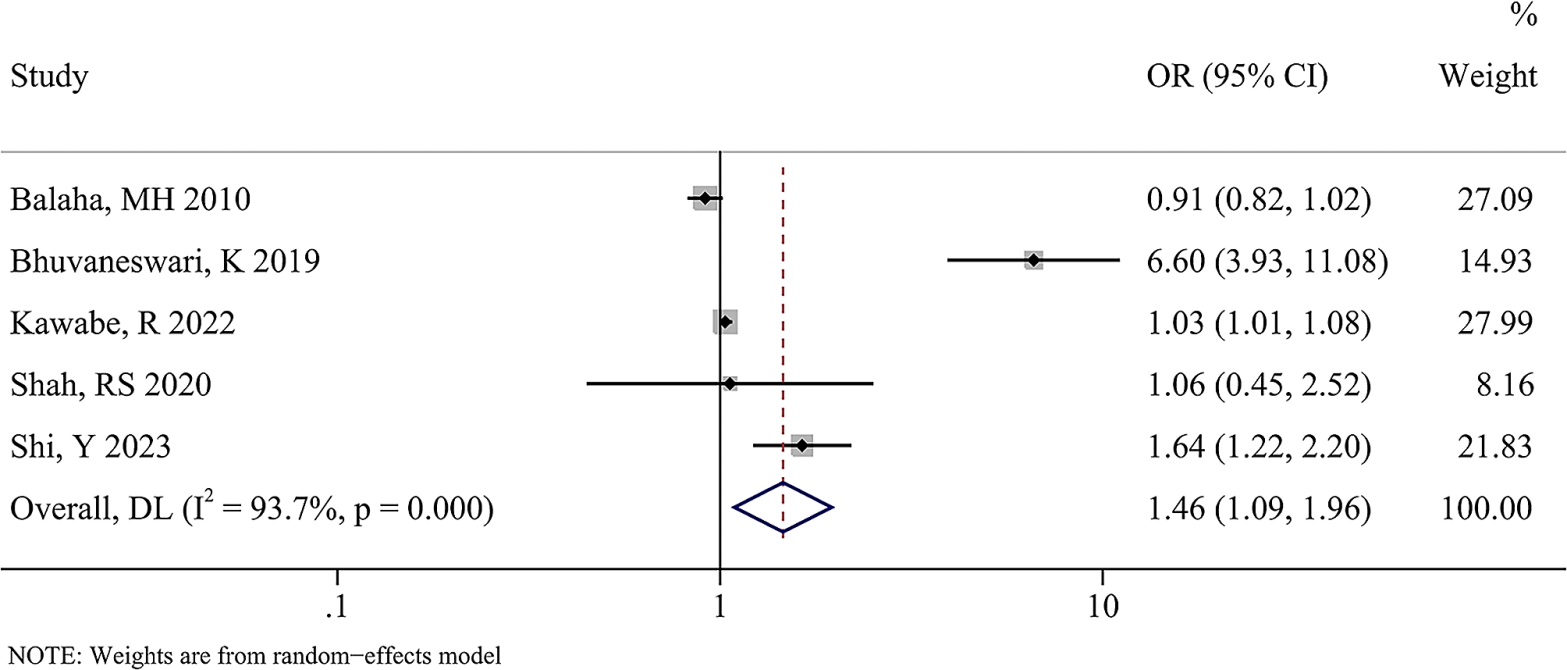
Forest plots showing the association between physical activity and the risk of premenstrual syndrome

The link between physical activity level and the risk of premenstrual syndrome in college students was analyzed by meta-analysis. There was significant heterogeneity between the studies in terms of both outcomes (I^2^ < 0.001%, *P* = .937). The pooled results of the random effects model revealed that low physical activity levels in college students were significantly related to an increased risk of premenstrual syndrome (OR [95%CI] = 1.46 [1.09, 1.96], *P* = .011, Fig. 2).

### Results of subgroup analysis

**Fig. 3:**
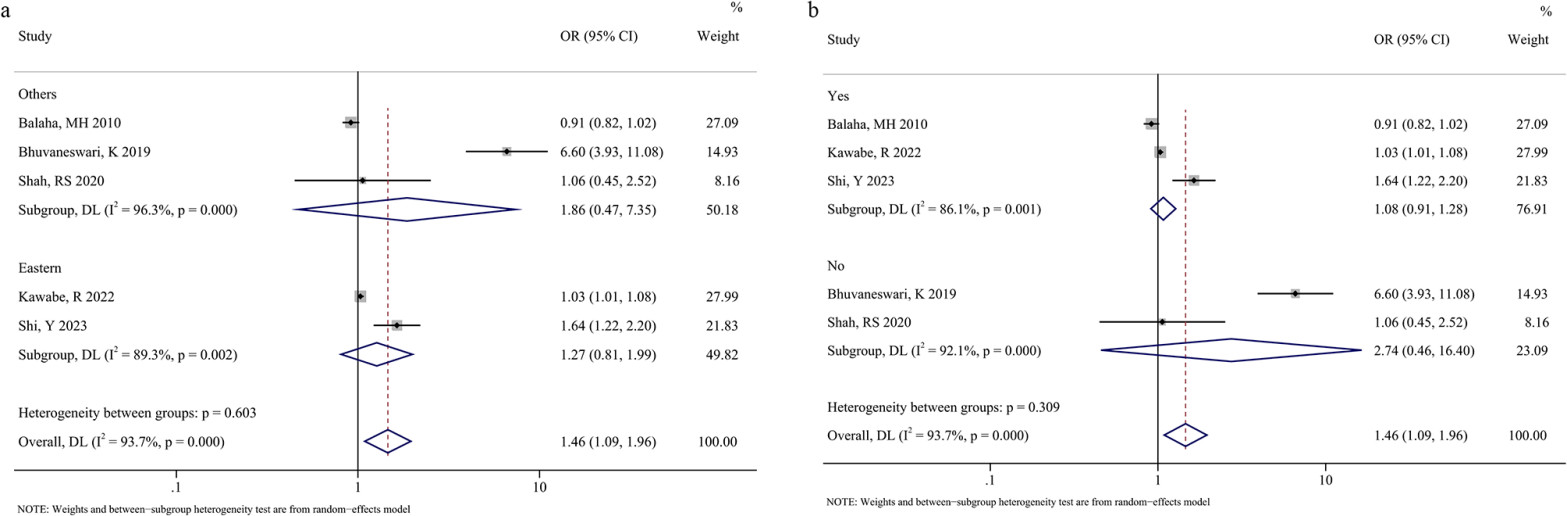
The results of subgroup analysis based on study region (**a**) and whether multi-factor correction was conducted (**b**)

To explore the source of heterogeneity, we performed subgroup analyses according to study region and whether multi-factor correction was conducted. By subgrouping according to study region, the pooled results of East Asia (OR [95%CI] = 1.27 [0.81, 1.99], *P* = .375) and the rest of Asia (OR [95%CI] = 1.86 [0.47, 7.35], *P* = .302) subgroups did not yield significant results (Fig. 3a; Table 2). By subgrouping according to whether the multifactor correction was conducted, the pooled results of the multifactor correction (OR [95%CI] = 2.74 [0.46, 16.40], *P* = .270) and non-correction (OR [95%CI] = 1.08 [0.91, 1.28], *P* = .401) subgroups also did not exhibit significant results (Fig. 3b; Table 2). Collectively, neither the study area nor multifactor correction influenced heterogeneity.

**Table 2:** Outcomes of subgroup analyses

Outcomes	No. of studies	OR (95%CI)	*P*_A_	Heterogeneity test	
				*P*	I^2^ (%)
**All**	5	1.46 (1.09, 1.96)	0.011	< 0.001	93.7
**Adjusted**					
No	2	2.74 (0.46, 16.40)	0.270	< 0.001	92.1
Yes	3	1.08 (0.91, 1.28)	0.401	0.001	86.1
**Region of** Asia					
Eastern	2	1.27 (0.81, 1.99)	0.375	0.002	89.3
Others	3	1.86 (0.47, 7.35)	0.302	< 0.001	96.3

*P*_*A*_: *P* value for test of the association

### Results of sensitivity analysis

**Fig. 4:**
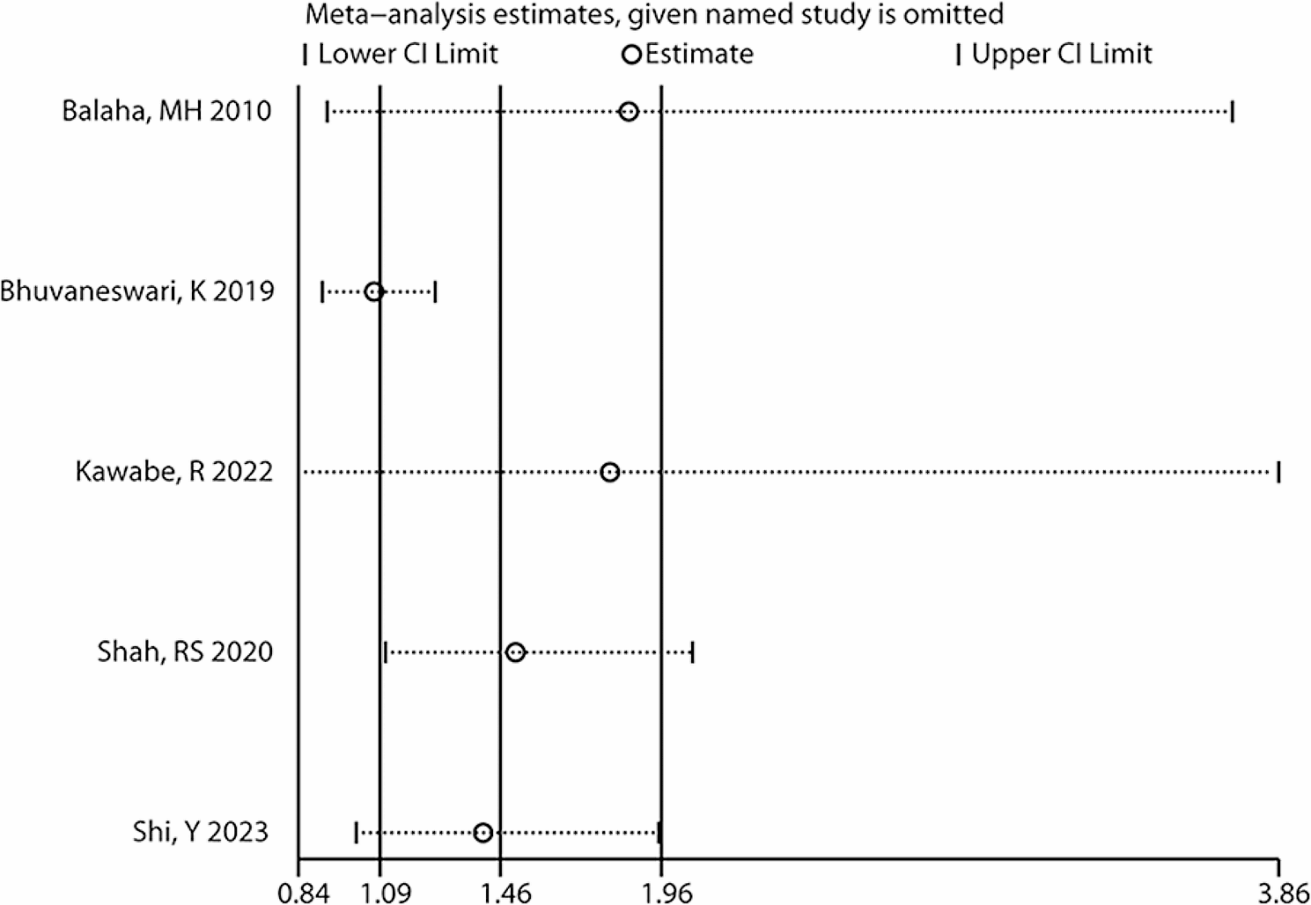
Sensitivity analysis showing the effect of a single study on the pooled results

We conducted a sensitivity analysis to explore whether the pooled results were influenced by a single study. Through the leave-one-out approach, the changes in pooled results ranged from OR (95% CI) = 1.07 (0.91, 1.26) to 1.86 (0.93, 3.72), indicating poor stability (Fig. 4). Excluding studies by Balaha et al [24], Bhuvaneswari et al [12], and Kawabe et al [18], the pooled results of the remaining studies were not significant.

### Publication bias

**Fig. 5:**
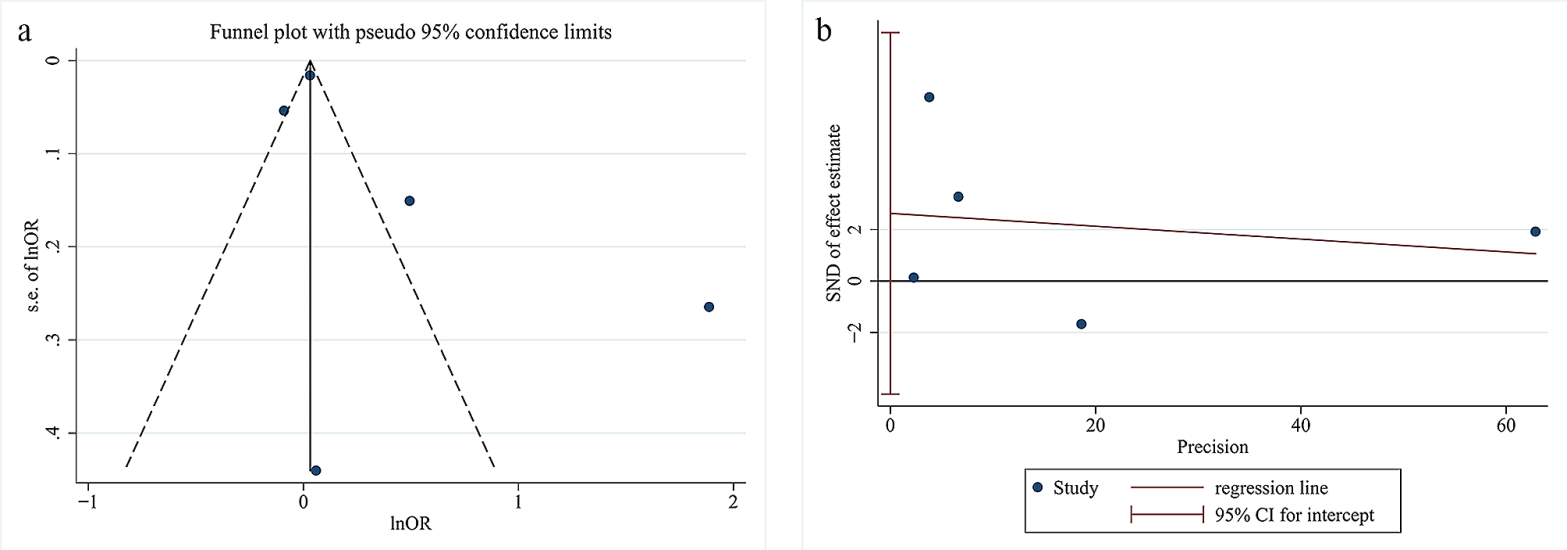
Publication bias results determined by funnel plot (**a**) and Egger’s test (**b**)

We further evaluated the publication bias. The funnel plot showed poor symmetry in the distribution of scatter points (Fig. 5a). However, the results of Egger’s test did not show a significant publication bias among the included studies (*P* = .319, Fig. 5b).

## Discussion

Premenstrual syndrome is a global public health problem that affects over 50% of women of reproductive age, including college students [26]. The burden of premenstrual syndrome is significant among female college students because it affects their mental health, academic performance, and relationships with other students [27, 28]. Several studies have revealed a link between physical activity and premenstrual syndrome; however, the findings are inconsistent [29]. In this meta-analysis, we included five studies to analyze the associations between physical activity and premenstrual syndrome and found that low physical activity levels increased the risk of premenstrual syndrome among female college students.

The exact causes of premenstrual syndrome are not fully understood, but it is essentially a neuro‑endocrine disorder in which neurobiological vulnerability plays a role in normal fluctuations in sex hormone levels during the menstrual cycle. These hormonal changes can affect the function of neurotransmitters or neuropeptides in the brain, which in turn contribute to the emotional and physical symptoms associated with premenstrual syndrome [30]. Women with premenstrual syndrome have an abnormal luteal phase in serotonin function compared with those without symptoms [31]. Considering the side effects of drug treatments, non-pharmacological treatment such as regular physical activity has been recognized as an approach to ease the symptoms of premenstrual syndrome. Physical activity can influence the risk of premenstrual syndrome through a variety of psychosocial and biological mechanisms. Physical activity can also reduce levels of steroid hormones such as estradiol and cortisol [32] and it may affect the function of sex hormones by altering the sensitivity of target tissues to these hormones [33]. Additionally, physical activity including resistive exercise, can improve muscle oxygenation, elevate neurotrophic factors and β-endorphin levels, induce neuroimmunomodulation effects, positively modulate hypothalamic pituitary gonadal hormone levels, improve the serotonin system, and alter mental and emotional states [34, 35]. These mechanisms may also support the inverse relationship between physical activity and the risk of premenstrual syndrome among female college students.

It’s worth noting that inositol has been shown to be effective in many aspects of women’s lives, from infertility to premenstrual syndrome. The role of inositol, insulin-sensitizing compounds of promising efficacy, in exercise physiology should not be overlooked, while its actions on the muscle and nervous system may further influence the premenstrual syndrome in the context of the effects of physical activity [36]. In some pathologic conditions, such as adenomyosis [37] or uterine fibroids [38] may exacerbate premenstrual syndrome in some women. The role of physical activity interventions in non-pharmacologic treatments with relevant prevalent populations deserves further exploration and may also be able to influence premenstrual syndrome by improving the reproductive health of women with the condition. In addition, studies in polycystic ovary syndrome (PCOS) have revealed the impact of obesity and insulin sensitivity on patients, and these factors have also been associated with physical activity in previous studies [39]. Further, physical activity-based interventions may have important and far-reaching implications for the reproductive health, with particular attention to issues related to early infertility and reproductive poor outcomes in terms of further prognosticating the risk of menopause [40].

This meta-analysis is the first to investigate the association between physical activity and premenstrual syndrome among female college students and provides the latest research evidence. Moreover, the publication bias test revealed that the combined results were more reliable. Despite these advantages, we also address the shortcomings and future research directions of our study. First, the included studies exhibited significant heterogeneity, and the subgroup analysis failed to identify significant influencing factors. Factors such as the intensity and type of physical activity as well as the graduate status of the participants might have an impact on heterogeneity. Owing to substantial differences in the information provided by the included studies, it was not possible to quantitatively analyze the effects of these factors on heterogeneity. Thus, future studies should establish unified outcome evaluation criteria and quantitative measurement standards for physical activity levels, as well as analyze the impact factors of physical activity. This will facilitate a more accurate evaluation of the association between physical activity and premenstrual syndrome and explore whether exercise interventions can prevent premenstrual syndrome. Second, all included studies were essentially cross-sectional in nature and susceptible to multiple confounding factors, which might affect the objectivity of the results. Additionally, the results of cross-sectional studies cannot determine causal relationships. Finally, the number of included studies was small as well as all from Asia, and the sensitivity analysis indicated poor stability of the meta-analysis results. More high-quality studies with large sample sizes are required to verify the stability and extrapolation of the results.

In conclusion, a high level of physical activity was associated with a reduced risk of premenstrual syndrome among female college students. This study recommends that healthcare professionals realize the risk of premenstrual syndrome and provide comprehensive services to college students. Moreover, providing awareness and education about the menstrual cycle and premenstrual syndrome is crucial for promoting open conversations and addressing issues related to menstruation among college students, which can reduce stigma and misconceptions surrounding menstruation and premenstrual syndrome and improve health care. In addition, measures based on exercise and physical activity interventions should be further emphasized for the prevention and clinical significance of associated symptoms. Meanwhile, universities should raise awareness among students and encourage them to engage in regular physical activity or establish effective coping measures.

### Electronic supplementary material

Below is the link to the electronic supplementary material.

Supplementary Material 1

Supplementary Material 2

Supplementary Material 3

Supplementary Material 4

## Data Availability

Datasets are available through the corresponding author upon reasonable request.

## Abbreviations

AHRQ: Agency for Healthcare Research and Quality
CI: confidence interval
OR: odds ratio
