# Supplementary material for: Association between physical activity and risk of premenstrual syndrome among female college students: a systematic review and meta-analysis
Source: BMC Womens Health. 2024 May 23;24:307. doi: 10.1186/s12905-024-03147-3 (PMC11112772; doi:10.1186/s12905-024-03147-3)
Supplement: Supplementary file 1 — Supplementary Material 1 [file 12905_2024_3147_MOESM1_ESM.docx]

**Supplementary Table 1** The search strategy of PubMed database

| Search | Query | Items found |
| --- | --- | --- |
| #1 | "premenstrual syndrome"[MeSH Terms] OR "premenstrual syndrome"[All Fields] | 5053 |
| #2 | "premenstrual dysphoric disorder"[MeSH Terms] OR "premenstrual dysphoric disorder"[All Fields] | 1096 |
| #3 | #1 OR #2 | 5243 |
| #4 | ("college"[tiab] OR "colleges"[tiab] OR ("universiti"[tiab] OR "universities"[MeSH Terms] OR "universities"[tiab] OR "university"[tiab])) AND ("students"[MeSH Terms] OR "students"[tiab] OR "student"[tiab]) | 117936 |
| #5 | "exercise"[MeSH Terms] OR "exercise"[tiab] OR "physical activity"[tiab] | 533434 |
| #6 | #3 AND #4 AND #5 | 22 |
