# Supplementary material for: Association between physical activity and risk of premenstrual syndrome among female college students: a systematic review and meta-analysis
Source: BMC Womens Health. 2024 May 23;24:307. doi: 10.1186/s12905-024-03147-3 (PMC11112772; doi:10.1186/s12905-024-03147-3)
Supplement: Supplementary file 2 — Supplementary Material 2 [file 12905_2024_3147_MOESM2_ESM.docx]

**Supplementary Table 2** The search strategy of Web of Science

| Search | Query | Items found |
| --- | --- | --- |
| #1 | (Premenstrual Dysphoric Disorder) OR (premenstrual syndrome) (All Fields) | 2950 |
| #2 | (College OR university) AND (students OR student) (All Fields) | 772019 |
| #3 | physical activity OR exercise (All Fields) | 989245 |
| #4 | #1 AND #2 AND #3 | 48 |
