# Supplementary material for: Association between physical activity and risk of premenstrual syndrome among female college students: a systematic review and meta-analysis
Source: BMC Womens Health. 2024 May 23;24:307. doi: 10.1186/s12905-024-03147-3 (PMC11112772; doi:10.1186/s12905-024-03147-3)
Supplement: Supplementary file 3 — Supplementary Material 3 [file 12905_2024_3147_MOESM3_ESM.docx]

**Supplementary Table 3** The search strategy of Embase database

| Search | Query | Items found |
| --- | --- | --- |
| #1 | ('premenstrual dysphoric disorder'/exp OR 'premenstrual dysphoric disorder') | 1821 |
| #2 | ('premenstrual syndrome'/exp OR 'premenstrual syndrome') | 5796 |
| #3 | #1 OR #2 | 6750 |
| #4 | ('college'/exp OR college OR 'university'/exp OR university) AND ('students'/exp OR students OR 'student'/exp OR student) | 362765 |
| #5 | ('physical activity'/exp OR 'physical activity' OR 'exercise'/exp OR exercise) | 906943 |
| #6 | #3 AND #4 AND #5 | 96 |
