# Supplementary material for: Association between physical activity and risk of premenstrual syndrome among female college students: a systematic review and meta-analysis
Source: BMC Womens Health. 2024 May 23;24:307. doi: 10.1186/s12905-024-03147-3 (PMC11112772; doi:10.1186/s12905-024-03147-3)
Supplement: Supplementary file 4 — Supplementary Material 4 [file 12905_2024_3147_MOESM4_ESM.docx]

**Supplementary Table 4** Quality assessment of the included cross-sectional studies

| Study | A | B | C | D | E | F | G | H | I | J | K | Total Scores |
| --- | --- | --- | --- | --- | --- | --- | --- | --- | --- | --- | --- | --- |
| Balaha, MH 2010 | 1 | 1 | 1 | 0 | 0 | 0 | 1 | 1 | 1 | 1 | 0 | 7 |
| Bhuvaneswari, K 2019 | 1 | 1 | 0 | 1 | 0 | 1 | 0 | 0 | 1 | 1 | 0 | 6 |
| Kawabe, R 2022 | 1 | 1 | 1 | 0 | 0 | 0 | 1 | 1 | 1 | 1 | 0 | 7 |
| Shah, RS 2020 | 1 | 1 | 0 | 1 | 0 | 0 | 0 | 0 | 1 | 1 | 0 | 5 |
| Shi, Y 2023 | 1 | 1 | 1 | 0 | 0 | 0 | 1 | 1 | 1 | 1 | 0 | 7 |

A: Define the source of information (survey, record review);

B: List inclusion and exclusion criteria for exposed and unexposed subjects (cases and controls) or refer to previous publications;

C: Indicate time period used for identifying patients;

D: Indicate whether or not subjects were consecutive if not population-based;

E: Indicate if evaluators of subjective components of study were masked to other aspects of the status of the participants;

F: Describe any assessments undertaken for quality assurance purposes (e.g., test/retest of primary outcome measurements);

G: Explain any patient exclusions from analysis;

H: Describe how confounding was assessed and/or controlled;

I: If applicable, explain how missing data were handled in the analysis;

J: Summarize patient response rates and completeness of data collection;

K: Clarify what follow-up, if any, was expected and the percentage of patients for which incomplete data or follow-up was obtained.
